# Essays on Theoretical and Practical Surgery

**Published:** 1846-04

**Authors:** 


Saggi di Chirurgia Teorico—Pratica di *Giuseppe Bresciani de Borsa,* Verona, 1843. Osservazioni Teorico-Pratiche. Di *G. Bresciani de Borsa.* Verona, 1844.

Essays on Theoretical and Practical Surgery. By *Joseph Bresciani de Borsa.* Pp. 448. Verona, 1843. Theoretical and Practical Observations. By *J. Bresciani de Borsa.* Pp. 42. Verona, 1844.

It is not often that works on Medicine and Surgery by Italian practitioners come to our hands; and, judging from the specimen we have before us, we regret much that we do not see more of them in this country. Dr. Bresciani de Borsa is in large private practice in Verona, and is surgeon to the general and lying-in hospitals of that city. The present works, in continuation of former publications, contain numerous cases in surgery, accompanied with appropriate observations. These latter, manifest a more extensive acquaintance with the foreign literature of the profession (always excepting the orthography of proper names) than we usually find in continental writers. We will proceed to lay a few extracts before our readers.

*The Cæsarian Operation.*—Two cases are detailed in which the author performed this formidable operation; the one terminating successfully as regards mother and child, the other being followed by death to the parent. The author was called to the first of these, *Angelina de Mori,* ætat. 20, 4 July, 1844; severe labor-pains had existed for two days, and had now become so violent as to threaten rupture of the uterus. The pelvis was much distorted by rickets, tilting the fundus uteri over the edge of the right ileum and abridging the aperture, so as to render the passage of an unmutilated child impossible. The obliquity of the uterus, indeed, was such that the author believes it alone would have justified the operation. The child being still alive, embryotomy, even if practicable, would have been, according to the notions of morals and religion prevalent in Italy, inexcusable. The operation was completed in two minutes, and the wound in the parietes brought together by five or six twisted sutures. By the 21st, notwithstanding peritonitis, requiring bleeding, &c. had supervened on the operation, and the woman being subsequently thrown into febrile action by a severe fright, the wound had quite cicatrized. An attack of phlegmasia dolens next came on, and as soon as she had recovered from this, the house in which she resided took fire, and she was carried through the flames on the 43rd day after the operation. Notwithstanding all this she did well.

The second case occurred in a dwarf woman, 32 years of age. The description given of her deformed person is such, that one is surprised how any human being could have had connexion with her. Her pelvis resembled that of a child, its antero-posterior diameter not exceeding an inch and a half. A live child was speedily extracted. The poor creature bore the operation with heroic firmness. "During its continuance she kept her face covered, and from her utter silence seemed as if dead; but as soon as it was completed, a crooked arm was stretched out from beneath the clothes towards the operator, and she was heard to say, 'Doctor, now you have finished the operation, *pray give me a pinch of snuff*.' Had I and so many others not been present at this exhibition of Spartan firmness, it could never have been believed, and yet the fact is indubitable." She died a week after the operation during a recurrence of a broncho-pneumonia, to which she had been subject at intervals for some months. On examination after death no inflammation of the uterus, peritoneum, or abdominal viscera, was discovered.

As might be expected in a country where the power of the Catholic Church is so predomidant, Dr. B. does not hesitate to recommend the Cæsarian Section as a preferable procedure to the destruction of a living child, and alludes with pious horror to the almost murderous practice of us English, who look upon the saving and baptising a child (he informs us as to one of the children he rescued he lost no time in baptising it and bestowing his own name and that of Julius Cæsar upon it) who has never yet seen the light as a very secondary matter to the adding additional risk to the dangerous condition of its parent. However, we are pleased to find that, even in that part of the world, the *induction of premature labour* at the seventh month has become a recognized operation. Two cases are here related in which it was practised: one, on account of the presence of convulsions and coma; and the other, in consequence of the contracted state of the pelvis. In both, the membranes were punctured, and the labour-pains commenced in an hour afterwards.

*Belladonna in Strangulated Hernia.*—"Before I was accustomed to apply Belladonna I resorted to the operation for the relief of strangulated hernia much more frequently than I do now. I am now able to enumerate, both in my hospital and private practice, a long series of cases in which the reduction of the hernia by the taxis, aided by this medicament, has been accomplished. I will not endeavour to explain the manner in which it acts upon the protruded or neighbouring parts, for I can only hazard hypotheses; I will not say with some that it relaxes the tendon of the external oblique, and the internal ring of the inguinal canal, as it expands the pupil: but will confine myself to the indisputable fact, that since I have employed the belladonna, I much oftener succeed in reducing the hernia without having recourse to the knife.

"I employ this formula. ℞. Ext. Belladon. ʒij. Axung. ℥ij. M. bene. I anoint the tumour at least every half hour. After a while the pain, and then the tension of the sac, diminish, and the possibility of reducing the hernia by the taxis occurs. The various other means usually employed, as bleeding, baths, cold, tobacco, &c. are not to be neglected; and, although these will frequently not succeed alone, they will do so in many cases when aided by the belladonna."

*Aneurism of the Brachial Artery after Venæsection.*—Dr. Bresciani de Borsa relates cases in which this was successfully cured by compression made by means of an instrument invented by Prof. Signorini, consisting of two semicircular limbs united by a screw which regulates the amount of pressure which they exert at their padded extremities, applied at opposite sides of the limb to be compressed. A plate is given. The author strongly deprecates the median-basilic vein being chosen for the purposes of venesection, for he has known the brachial artery wounded by even accomplished practitioners during a sudden movement of the patient. He lays down as general rules—" 1. Avoid if possible opening the median-basilic. 2. If this vein is prominent, and the median-cephalic, the radial, and the cubital are hardly visible, nevertheless choose one of these last, though its calibre may be smaller. If it is skilfully opened enough blood will be obtained. 3. If the median-basilic is alone visible, and none of the others can be seen or felt, which is however rarely the case, bleed in the middle of the fore-arm, the hand, or the foot. 4. If the median-basilic is the only practicable vessel, which is scarcely possible, conduct the operation slowly, allowing only a sufficiency of the lancet to penetrate the vein to project beyond the fingers, and then, if any sudden movement of the patient does occur, the fingers would prevent the penetration of the instrument through the farther side of the vein." Certainly many practitioners exhibit a fool-hardiness in bleeding which should be deprecated; but our author's fears seem somewhat exaggerated. The accident is usually produced by the perpendicular thrust of the uninstructed pupil; but when the vein is prominent, and the lancet carried horizontally, surely there is little or no danger of piercing through the vessel.

" *Mode of curing obstinate old Ulcers.*—When I have met with very old ulcers, especially those of the leg, which resist every other method of treatment, I have obtained their sound cicatrization by instituting, by means of caustic potass, a new ulcer in the vicinity. I make in a piece of adhesive plaister a hole, somewhat smaller in size than I wish the artificial ulcer to be, and then apply it at one or two finger's breadth from the old sore. Caustic potass is rubbed on this space until an eschar is formed; and I have constantly observed that, during the consequent inflammatory and suppurative processes, the old solution of continuity, which had so obstinately resisted treatment, has closed up, and the cicatrix has in general continued sound.

" If the healed ulcers had resulted from a disordered constitution, to the appropriate internal treatment, I add either an issue in some usual spot, or place a small portion of wax in the artificial ulcer itself when nearly healed, so as to convert it into a common issue, which contributes much to efficient treatment, as by such prudential precaution I have never seen any mischief produced in the constitution of those who had long been subject to obstinate ulcers. If the ulcer was produced by a traumatic cause, after it has become healed the artificial one may also be cicatrized as soon as possible without any injury resulting.

" In my practice I have cured more than a hundred cases in this manner, and many instances have occurred in the hospital where I have cured ulcers of twenty or thirty years' standing."

*Extraction of a musket-ball which had been lodged in the orbit for twenty-four years.*—*Felice*, an old soldier of one of Napoleon's armies, was, during an engagement, struck just above the left orbit by a musket-ball, but as a comrade fell dead at the same time at his side, he believed the ball had rebounded from the orbit and killed him. For more than 24 years after he was continually the subject of violent pains in the left eye and to attacks of cephalalgia—the eye itself projecting much from the orbit. The numerous surgeons under whose care he placed himself from time to time, believing his tale of the rebounding of the ball, afforded him little or no relief, and in 1837, he came to the hospital at Verona. The author, upon examining the case, came to the conclusion, that the projection of the eye could only be caused by the persistence of the foreign body in the orbit, for any exfoliation of bone which the blow might have caused would, in the course of so many years (the projection commencing soon after the accident), have been discharged or removed by absorption. A portion of bone was therefore at once removed from the orbit by the trephine. The track of the ball was found ossified, excepting at a small aperture, whence issued from time to time a little fluid. After the bone was removed, the ball was felt by means of a probe at the back of the orbit and removed by means of a forceps. The eye now retreated into the orbit, and after some weeks passed into a state of atrophy. The violent pains were quite relieved, and the patient died five years afterwards only, of pleuro-pneumonia. On examination, it was found that the cranial cavity had not been penetrated by the trephine, but opposite to where the bone had been removed was a deposit of osseous substance.

*Wound of the Bladder.*—A young man, in a scuffle with a companion, was stabbed by him in the hypogastric region with a knife. The instrument penetrated the bladder, but this viscus being distended by reason of his having been drinking, the wound occurred below the peritoneal fold. The urine became immediately diffused about the neighbouring parts, and as soon as he was brought to the hospital a catheter was introduced, through which a great deal of both blood and urine flowed. The external wound in the abdomen was closed by adhesive plaster; and, in the course of the cure, bleeding was practised. His recovery was rapid, and so complete, that he was enabled to be received a soldier. He died in a few months of some febrile affection, the cicatrix continuing sound.

Wounds of the bladder by cutting instruments are rare, for Larrey only mentions one case, although those arising from fire-arms are frequent. Samuel Cooper, however, mentions a case much resembling the above in his Dictionary.

*Small-pox in the Fœtus.*—A young woman, suffering from small-pox, was delivered of twins in the hospital, one of them having numerous finely-developed pustules, while the other was entirely free from the eruption. "What will the deniers of the possibility of contagion acting upon the fœtus *in utero* say to this case? I would explain the fact of the one child not becoming affected by the absence of predisposition."

"*Creosote in obstinate Ulcers.*—This application for indolent and obstinate ulcers seems to be going out of use. I do not know why, for, in my own practice, so useful do I find it, that I may call it a sovereign remedy. This is the formula I use—℞. *Creosote* gtt. vi., *Aq. font.* ℥iv. M. I increase the quantity gradually to 10 or 20 drops. Whoever has attended my hospital practice must have observed with what celerity a change in the pathological condition of old, foul, indolent, ulcers is brought about. It is decidedly the best flesh-producer *(sarcotico)* known in surgery in these morbid affections. Has the remedy some specific action on the capillaries? for in even 20 hours I have seen a foul surface covered by luxuriant granulations, and old, indolent ulcers become benign and active. Certainly the surgeon must always reserve the application for old and indolent ulcers; for if he orders it in active, inflammatory, and painful ones, great mischief will result; and I believe it has fallen into disrepute, because it has so frequently been prescribed in conditions which contra-indicated its employment."

*Urinary Fistulæ.*—Dr. Bresciani de Borsa states that he has tried the plan of leaving a catheter in the bladder, and that of allowing the urine to pass along the canal; but finds there are strong objections to both. The constant presence of a catheter induces chronic cystitis, inflammation of the neck of the bladder, or even a fistula of the organ. On the other hand, left to itself, the urine prevents the union of the edges of the fistulous opening. A mixt method is the best, and this consists in passing, the instant the urgency for voiding urine is felt, a conical silver catheter cut off at the end, so as to leave the apex open, and thus withdraw the urine from the bladder without allowing it to come in contact with the fistula, and yet avoid the dangers consequent upon the retention of the instrument in the bladder.

### Lithotomy.

Nearly one-half the work is occupied with this subject. The author details at great length the symptoms, modes of detecting, and prognosis of stone in the bladder. After dwelling minutely and ably upon the anatomy of the parts concerned, and describing all the various operations which have been devised for the removal of the calculus, he presents us with an account of his own mode of procedure, which he states to have been attended with an amount of success which seems to us almost incredible, much as we are disposed to rely upon the veracity of so industrious and candid an observer. This operation is, in fact, a modification of that of which Signor Manzoni, of Verona, published an account in 1808; and Dr. de Borsa declares that, of *one hundred cases operated upon by that practitioner and himself, only one has died;* and that from causes irrespective of the operation! Manzoni's operation consisted in cutting into the spongy portion of the urethra only, and then dilating the bulbous and prostatic portions sufficiently with the finger to admit of the introduction of the forceps and the removal of the stone. Dr. B., however, judging it more reasonable to cut into the more dilatable portion of the urethra, carries his incision from the bulb to the prostate, and even unavoidably scarifies the latter when it advances more than usually forwards. As the admission of such an operation depends upon the dilatability of the prostate being satisfactorily proved, the author advances several reasons for this, founded on the nature of its anatomical structure, and cites various facts observed by himself and others. He then details the various steps of his operation.

" Having placed the patient in the usual position, (it is much preferable to retain him in this by means of assistants than by ligatures, for the mere ceremony of adjusting these, causes a great dread to the patient which may alone suffice to induce a low and fatal form of fever), introduced the catheter, and made a sufficiently large external incision, I open, with a small, lancet-pointed, double-edged, strong, scalpel, the whole of the membranous portion of the urethra, so as to expose the instrument to the extent of about 10 lines, in doing which it may in some cases easily happen that the apex of the prostate is also cut, although in the case in which the patient died it was found entire. I now take hold of the handle of the catheter, and, passing my left fore-finger into the wound, feel the groove exposed; and as others would pass some form of gorget through the prostatic portion of the urethra and neck of the bladder, I only introduce my finger into the bladder, being certain it never can make a false passage, since I keep it in contact with the metallic instrument. I take care, nevertheless, not to pass my finger along the groove, because I should then thrust it against the internal or posterior angle of the wound, and then against the great bulk of the prostate. Scarcely do I touch the groove before, instead of following it, I pass my finger upon the right side of the staff (as regards the patient) and carry it quietly and without any obstacle into the bladder. One of the advantages of this modification is, that it enables me, in most cases, to come at once in contact with the stone.

"I then remove the staff, still however retaining my finger within the track of the incision, and gently moving it about in a semi-rotatory manner, effect a much greater dilatation of the prostatic urethra and neck of the bladder. Next, I pass in the forceps behind my finger, and seize the stone. It has to pass along a track of only from 12 to 15 lines, or even less, since the inverse cone formed by the forceps approximates the neck of the bladder and urethral aperture, for which reason the space is shorter and more easily dilated. I remove the stone with two, or at most three, semi-rotations, performed with circumspection and care along an axis, which, commencing at the centre of the bladder, should pass through its neck, and following the centre of the prostatic urethra, terminate in the centre of the perineal aperture.

"I am in general not more than a minute after opening into the urethra before I have extracted the stone, and the operation has always succeeded in my hands. Safety, simplicity, and celerity, I have already said are desiderata of every operative process, and I am in a condition to prove that they attach to this one recommended by me. All I employ are simply a bistoury and the forceps—*simplicity*, surely, in comparison with the multiplicity of complicated instruments which has been recommended in the various and numerous modes of performing *Cystotomia*. By passing my finger along the staff, I secure that *safety* which is not usually a characteristic of operations for stone. It is related that even the celebrated Scarpa passed the gorget, which was looked upon as the palladium of his fame, in between the bladder and the rectum. The same thing has occurred to many otherwise skilful operators. * * * * * I am certain of not injuring either the pudendal artery, the prostatic venous plexus (so frequently in a varicose condition in the aged), the body of the bladder, or the rectum. Farther, by the preservation of the whole, or nearly the whole, of the prostatico-vesical canal, inflammations between the rectum and bladder are avoided, inasmuch as urinary infiltration is prevented, in consequence of the prostate not being divided. * * * * * The *rapidity* of the operation is shown by the fact, that instantly after I have opened the urethra I have the stone in my hand; and any one who has even once performed this operation, will bear witness to my assertion. I may observe that it seems almost impossible that so many operators, at all periods, have written so much, and cudgelled their brains to invent new operative procedures for Lithotomy by deep prostatico-cystic incisions, while, with a simple *urethrotomy*, the desired end may be obtained, as I can prove by so many cases."

The author next enters into a detailed historical account of the twenty or thirty modifications of the operation of lithotomy which have been at various periods devised, exhibiting the particulars in which his own may be advantageously compared with these ; and terminates his Essay with a Report of the discussion upon the *dilatability of the prostatic portion of the urethra* which took place in the Medical Section of the Scientific Congress held at Padua two or three years since. At this, several eminent practitioners expressed doubts of the feasibility and safety of his proceedings ; but he truly observed, that facts are far beyond mere theoretical objections. Upon it being insinuated by some that success attendant upon the removal of small stones ought not to confer approval upon a method, Dr. B. replied by exhibiting large calculi which he had removed. He stated, however, that neither this or any other form of operation can be universally practised, and that the bi-lateral operation of Dupuytren is best adapted for stones of great magnitude, and the hypogastric operation when this is excessive. So, too, where the prostate is much diseased, the hypogastric operation may be more eligible. The dilatability of the prostatic urethra, independently of incision or laceration, however, was considered so doubtful by many, that a Commission was appointed to conduct some experiments on the dead body. To conclusions drawn from such a source, the author repeatedly enters his protest. He maintains that the dilatability of the prostate in the living body is mainly due to its vital properties, and that laceration is far more likely to be produced in the mere organic structure of the dead body. However, the Commission was formed, and he consented to exhibit the celerity with which the finger and forceps might be introduced into the bladder, but declined allowing the soundness of his practice to depend upon conclusions drawn from circumstances so different to those prevailing in the living body. Here he only exercised a sound discretion. In the two instances experimented upon, upon examination afterwards, the prostate was found to be only slightly lacerated to the extent of two or three lines, although the forceps had been introduced and the stone removed by the aperture. This for the dead body ; but the author reiterates that, in the living subject, a division of even the apex of the prostate is exceptional and unessential, and that, in the only case in which death allowed of an examination being made, the part was found entire, as testified by competent witnesses, although the stone was 14 lines in diameter. He observes, moreover, of those who persist in maintaining he must lacerate the prostate in dilating it, that they must find great difficulty in explaining Manzoni's great success, who only opened into the spongy portion of the urethra, leaving entire, between the end of his incision and the apex of the prostate, the bulb and the membranous portion of the urethra, and extracting the stones without any lacerations whatever, and with uniform recoveries. The author quotes Dr. Willis's opinion of the suitableness of an operation conducted on these principles, an account of which he gives in his work on Stone, under the appellation of *Lithectasy* ;* but, as we find Dr. B. is a reader of the *Medico-Chirurgical Review*, we may as well inform him

---

* Med. Chir. Rev. April 1842, p. 490.

that the authority of Dr. Willis, whom he designates as "*l'illustra litotomista,*" is not of much weight here in this matter. He is doubtless not aware that physicians do not operate in this country; and the performing operations and the suggesting them are mighty different things. Dr. Willis' work on the Stone is a mere compilation. Other corroborative but incidental authorities are cited as recommendations of the practice advocated; but we have no space for adverting to them. The position the author occupies as one of the *élite* of the profession in his own country, his great experience, and evident candour, lead us to accept his statements as well-grounded, and to hope that they will be put to the test of experiment among ourselves.

These "Essays" form a valuable contribution to surgical practice and literature, and are abundantly illustrated by lithographs.

